# Prevalence of hypertension and its correlates in Lusaka urban district of Zambia: a population based survey

**DOI:** 10.1186/1755-7682-4-34

**Published:** 2011-10-05

**Authors:** Fastone M Goma, Selestine H Nzala, Olusegun Babaniyi, Peter Songolo, Cosmos Zyaambo, Emmanuel Rudatsikira, Seter Siziya, Adamson S Muula

**Affiliations:** 1 Department of Physiology, School of Medicine, University of Zambia, Lusaka, Zambia; 2 Department of Community Medicine, School of Medicine, University of Zambia, Lusaka, Zambia; 3 World Health Organization Country Office, Lusaka, Zambia; 4 School of Community and Environmental Health, Old Dominion University, Virginia, USA; 5 Department of Community Health, University of Malawi, College of Medicine, Blantyre, Malawi

**Keywords:** Hypertension, BMI, alcohol consumption, sedentary behaviour, fasting blood glucose level, Lusaka, Zambia

## Abstract

**Background:**

Hypertension is a leading cause for ill-health, premature mortality and disability. The objective of the study was to determine the prevalence and associated factors for hypertension in Lusaka, Zambia.

**Methods:**

A cross sectional study was conducted. Odds ratios and their 95% confidence intervals were calculated to assess relationships between hypertension and explanatory variables.

**Results:**

A total of 1928 individuals participated in the survey, of which 33.0% were males. About a third of the respondents had attained secondary level education (35.8%), and 20.6% of males and 48.6% of females were overweight or obese. The prevalence for hypertension was 34.8% (38.0% of males and 33.3% of females). In multivariate analysis, factors independently associated with hypertension were: age, sex, body mass index, alcohol consumption, sedentary lifestyle, and fasting blood glucose level.

**Conclusions:**

Health education and structural interventions to promote healthier lifestyles should be encouraged taking into account the observed associations of the modifiable risk factors.

## Background

The major non-communicable diseases (NCDs) which include cardiovascular diseases (CVD), cancers, chronic respiratory diseases and diabetes contribute immensely to mortality globally [1-3]. Hypertension, a major risk factor for CVD and renal diseases, significantly reduces life expectancy [4]. Myocardial infarction and stroke occur 2-3 times more often among individuals with untreated hypertension. In a study conducted in Kenya, Ogend'o et al [5] found that hypertension was the most common risk factor for acute myocardial infarction, followed by diabetes mellitus, smoking, infection, and alcohol. Indeed coronary heart disease (CHD) has emerged as the leading cause of death among older Africans [6].

Hypertension, like other NCDs, is associated with identifiable behavioural and biological risk factors [7-10]. The major risk factors include race, obesity, diabetes, age, sex, alcoholism, sedentary lifestyle, diet (including salt intake), and family history of hypertension. Some of these risk factors for hypertension are modifiable through lifestyle interventions or at least their effects ameliorated by lifestyle modifications and medical management. Public health and primary care intervention against NCDs in the low-income nations are often inadequate, despite the increasing prevalence rates of these diseases. Ezzati et al [11] have suggested that what were commonly referred to as "diseases of affluence" (e.g. diabetes, CVD) can no longer be categorized as low prevalence conditions in low income nations. In response, Maher et al [12] have argued that decreasing the chronic NCD burden requires a two-pronged approach, i.e. implementation of the multisectoral policies aimed at decreasing population-level risks for NCDs, and effective and affordable delivery of primary care interventions for patients with chronic NCDs. These goals can best be achieved when the prevalence of population-level risks such as hypertension, are known.

In order to contribute to the literature on hypertension globally, but more specifically in low-income nations, the present study was conducted mainly to estimate the prevalence of hypertension and explore the factors that are associated with the condition in urban Lusaka, Zambia.

## Methods

### Sample size and sampling

This was a secondary analysis of extant data. A comprehensive description of the survey methods of the original study has been reported elsewhere [13,14]. The survey used a modified WHO global surveillance initiative NCD-STEP 3 [15]. The sample size of 1928 study participants was powered enough to produce estimates for Lusaka district by sex and age.

A multi-stage cluster sampling technique was used to select study participants; in each selected constituency, one ward was selected. The number of Standard Enumeration Areas (SEAs) selected in each ward was proportional to its population size. A systematic random sampling method was used to select the SEAs. Within the SEAs, households were then systematically sampled in order to widely cover the area. All persons aged at least 25 years in the selected households were invited to participate in the survey.

### Ethical considerations

The University of Zambia (UNZA) Research Ethics Committee (REC) reviewed the survey protocol. Informed consent was obtained from each of the study participants. All entry forms were kept in the office of the Principal Investigator and completed questionnaires were only viewed by approved study personnel.

### Data collection

The WHO global surveillance initiative for NCD [15] which has three steps was used: Step 1 consists of a questionnaire, Step 2 is physical examinations, and Step 3 is biochemical examinations. The questionnaire was interviewer-administered. All these steps were conducted within the participants' homes. Interviewers included nurses and laboratory technicians. These had undergone a 5 days training in both administering the questionnaire and taking measurements as described by the World Health organization in the manual on the surveillance of NCDs [15].

#### Interviews

An interview schedule was used to obtain responses from the interviewees. The questionnaire was divided into the following sections among others: Demographic information, alcohol consumption, sedentary behaviour, height and Weight.

#### Blood pressure

The OMRON digital automatic BP monitor M4-1 (OMRON Healthcare Europe BV, The Netherlands) was used to measure the blood pressure of the participants. Three minutes of rest was given to the participant in between three successive readings of blood pressure. Although the three readings were different with the largest value being the first reading and the smallest being the third reading on average, these differed by no more than 2 mmHg of systolic blood pressure, and no more than 4.5 mmHg of diastolic blood pressure. We chose to take an average of the three reading, and not the average of the second and third readings as recommended by World Health Organisation [15] in order to increase the degrees of freedom for the mean.

#### Height

The Seca Brand 214 Portable Stadiometer (Seca gmbh & Co. kg Humburg, German) was used to measure the height of the participant. Height was measured without the participant wearing foot or head gear. Before the reading was taken, the participant was requested to have feet together, heels against the back board, knees straight, and look straight ahead. Height was recorded in centimetres.

#### Weight

Weight was measured using the Heine Portable Professional Adult Scale 737 (Seca gmbh & Co. kg Humburg, German). Participants were asked to stand still, face forward, and place arms on the sides of the body. Weight was recorded in kilograms.

#### Waist circumference

The Figure Finder Tape Measure was used to measure the waist circumference in centimetres. This measurement was taken in a private area. The midpoint between the inferior margin of the last rib and the crest of the ilium were marked using a tape measure. With the assistance of the participant, the tape measure was wrapped around the waist directly over the skin or light clothing. Just before the measurement was taken, the participant was requested to stand with their feet together, place their arms at their side of their body with the palms of their hands facing inwards, and breathe out gently.

#### Hip circumference

The measurement for hip circumference was taken in a private area immediately after the waist circumference. The Figure Finder Tape Measure was used in measuring the hip circumference in centimetres. The measurement was taken at the maximum circumference over the buttocks, after requesting the participant to relax the arms at the sides.

#### Heart rate

The heart rate was recorded simultaneously while taking blood pressure readings using the ORMRON digital automatic blood pressure monitor M4-1 (OMRON Healthcare Europe BV, The Netherlands).

#### Glucose and Cholesterol

Fasting glucose and total cholesterol were determined using an Accutrend GCT Meter Three-in-One System (Glucose, Cholesterol and Triglycerides) (Roche Diagnostics GmbH, Mannheim, German).

### Data entry

Two data entry clerks were trained to enter the data using Epi Data version 3.1. Data was double entered and validated. The data entry template had consistency and range checks embedded in it. The data entry clerks were trained and supervised by the Principal Investigator. The validated data was exported to SPSS version 14.0 for analysis.

### Data Analysis

The analysis included running cross-tabulations, bivariate, and multivariate logistic regression. A backward variable selection method in logistic regression was used to determine independent predictors for hypertension. Unadjusted odds ratios (OR) and their 95% confidence interval (CI), and adjusted odds ratios (AOR) and their 95%CI are presented. Body mass Index (BMI) was categorized as <18.5 kg/m^2 ^(lean), 18.5-24.9 kg/m^2 ^(normal), 25.0-29.9 kg/m^2 ^(over weight), and 30+ kg/m^2 ^(obese); waist-hip ratios was grouped into two: <1 (normal) and >1 (raised); A participant with blood pressure of more than 140/90 mmHg was classified as being hypertensive. Fasting glucose levels were grouped into hypoglycaemia (<3.3 mmol/L), normal (3.3-5.5 mmol/L), and impaired glucose tolerance (5.51-8.49 mmol/L) or diabetes (8.5 mmol/L or more); Heart rate was grouped as normal (60-90 beats per minutes), slow (<60 beats per minute) or fast (>90 beats per minute); and cholesterol levels were either normal (<5.2 mmol/L) or otherwise raised.

## Results

### Socio-demographic characteristics and prevalence of hypertension

A total of 1928 individuals participated in the survey, of which 33.0% were males. About half of the participants were of age 25-34 years (53.2%), and a third of the respondents had attained secondary level of education (35.8%). The prevalence for hypertension was 34.8% (38.0% of males and 33.3% of females). These results are shown in Table 1.

**Table 1:** Demographic characteristics and hypertension prevalence for the participants in Lusaka, Zambia

	Total	Male	Female
Factor	n (%)	n (%)	n (%)
**Age group (years)**			
25-34	1015 (53.2)	337 (53.7)	675 (52.9)
35-44	413 (21.6)	135 (21.5)	277 (21.7)
45+	481 (25.2)	156 (24.8)	323 (25.3)
**Sex**			
Male	634 (33.0)	-	-
Female	1288 (67.0)	-	-
**Education**			
None	408 (21.5)	76 (12.2)	330 (26.0)
Primary	276 (14.5)	61 (9.8)	214 (16.9)
Secondary	679 (35.8)	242 (38.8)	435 (34.3)
College/university	534 (28.1)	244 (39.2)	290 (22.9)
**Hypertension**			
No	1231 (65.2)	387 (62.0)	840 (66.7)
Yes	658 (34.8)	337 (38.0)	419 (33.3)

### Factors associated with hypertension

Factors associated with hypertension are presented in Table 2. Of the factors considered in bivariate analyses to be associated with hypertension, age, sex, education level, sedentary lifestyle, body mass index, waist-hip ratio, levels of cholesterol, fasting blood glucose, and heart rate were significantly associated with hypertension.

**Table 2:** Factors associated with hypertension

	Unadjusted	Adjusted
Factor	OR (95%CI)	AOR (95%CI)
**Age group (years)**		
25-34	1	1
35-44	0.83 (0.71, 0.98)	0.82 (0.69, 0.97)
45+	2.84 (2.44, 3.31)	2.75 (2.32, 3.25)
**Sex**		
Male	1	1
Female	0.90 (0.82, 1.00)	0.84 (0.74, 0.96)
**Education**		
None	1	-
Primary	0.97 (0.79, 1.19)	
Secondary	0.82 (0.70, 0.96)	
college/university	0.92 (0.78, 1.09)	
**Time usually spent sitting or reclining on a typical day (hours) **		
<1.5	1	1
1.5-3.4	1.00 (0.88, 1.14)	1.08 (0.92, 1.25)
3.5+	1.32 (1.16, 1.52)	1.21 (1.03, 1.43)
**Body Mass Index (kg/m^2^)**		
<18.5	1	1
18.5-24.9	0.70 (0.60, 0.83)	0.78 (0.64, 0.94)
25.0-29.9	1.15 (0.96, 1.38)	1.32 (1.07, 1.64)
30+	2.48 (1.99, 3.08)	2.25 (1.73, 2.92)
**Alcohol consumption**		
Yes	1	1
No	0.91 (0.82, 1.01)	0.87 (0.76, 0.99)
**Cigarettes smoking**		
Yes	1	-
No	0.97 (0.81, 1.17)	
**Waist-Hip ratio**		
≤ 1	1	-
>1	1.76 (1.10, 2.81)	
**Cholesterol (mmol/L)**		
<5.2	1	-
5.2+	1.30 (1.14, 1.48)	
**Fasting blood glucose level (mmol/L)**		
3.3-5.5	1	1
<3.3	0.60 (0.49, 0.73)	0.70 (0.55, 0.88)
>5.5	2.86 (2.02, 4.06)	1.75 (1.17, 2.62)
**Heart rate (beats per minute)**		
60-90	1	-
<60	0.77 (0.63, 0.96)	
>90	1.04 (0.78, 1.38)	

In multivariate analysis, the factors associated with hypertension were: age, sex, body mass index (BMI), alcohol consumption, sedentary lifestyle, and fasting blood glucose. Compared to respondents in the age group 25-34 years, respondents aged 35-44 years were less likely (AOR = 0.82, 95%CI [0.69, 0.97]), and those aged 45 years or older were more likely (AOR = 2.75, 95%CI [2.32, 3.25]) to have hypertension. Female respondents were 16% (AOR = 0.84, 95%CI [0.74, 0.96]) less likely to have hypertension compared to males. Compared to respondents with BMI of <18.5 mmol/L, those with BMI of 25+ mmol/L were more likely to have hypertension (AOR = 1.32 (95%CI [1.07, 1.64]) for 25.0-29.9 mmol/L BMI, and AOR = 2.25 (95%CI [1.73, 2.92]) for 30+ mmol/L BMI; and respondents with 18.5-24.9 mmol/L BMI were 22% (AOR = 0.78, 95%CI [0.64, 0.94]) less likely to have hypertension. Participants who did not consume alcohol were 13% (AOR = 0.87, 95%CI [0.76, 0.99]) less likely to have hypertension compared to those who consumed alcohol. Compared to participants who spent less than 1.5 hours sitting or reclining on a typical day, those who spent 3.5 hours or more were 21% (AOR = 1.21, 95%CI [1.03, 1.43]) more likely to have hypertension. Compared to participants who had normal (3.3-5.5 mmol/L) glucose levels, those who were hypoglycemic (<3.3 mmol/L) were 30% (AOR = 0.70, 95%CI [0.55, 0.88]) less likely to have hypertension, and those who had impaired glucose levels or were diabetic (>5.5 mmol/L) were 75% (AOR = 1.75, 95%CI [1.17, 2.62]) more likely to have hypertension.

## Discussion

In a study among adults in urban Lusaka, Zambia, the prevalence for hypertension was 34.8% (38.0% of males and 33.3% of females). Delas et al [16] reported for a multi-setting data that the prevalence of hypertension in sub-Saharan Africa ranges from 6% to 48%. Our prevalence estimates are higher than those reported from South Africa of 25.5% among females and 21.6% for males [17], Uganda (22.0%) [18], and Eritrea (16%) overall [19,20]. Compared to the prevalence of hypertension reported in Zimbabwe [21], our estimates are higher for males (38.0% for Lusaka vs. 26% for Zimbabwe) and lower for females (33.3% for Lusaka vs. 41% for Zimbabwe).

We found that being overweight or obese, having raised cholesterol, older age group, sedentary lifestyle, use of alcohol, and having impaired glucose level were associated with hypertension. These findings are consistent with those from previous studies in other African countries [18,22-24]. In South Africa, van Rooyen [22] found that age was positively correlated with blood pressure. In Cameroon, Shey Wiysonge et al [23] reported a significant association between obesity and hypertension. In Egypt, El-Shafei et al [24] reported that age, and elevated BMI were significantly associated with an increased risk of essential hypertension. In Uganda, hypertension was found to be associated with increasing age, high BMI, elevated glucose, and alcohol use [18,25].

We found that being male was associated with hypertension; this is consistent with findings from Tanzania [26]), but different from what Wamala et al [25] have reported from Uganda where females were more likely to have hypertension than males. The clustering of the risk factors for hypertension (obesity, male, alcohol, glucose intolerance) is important to note as public health interventions aimed at reducing one (of the risk factors) may also impact the prevalence of the other.

## Limitations of the current study

Though the study design provides reliable and valid information, the study may have some limitations. The survey was done in Lusaka district, and hence the results can only be generalized to the sampled population. Some information was missing, especially for total cholesterol. Most of the test strips for total cholesterol could not produce results. We did not have reliable information on the number of household members of age 25 years or older in order to enable us to compute response rates. Therefore, we could not compute weights that could have been used in the analysis. Our findings may be biased to the extent that non-respondents differed from those that participated in the survey. However, we are unable to suggest the direction of the bias. Some study factors in our survey were obtained through self-reports, and as in all such studies, both inadvertent and deliberate reporting is a concern, more so that we obtained personal identifiers.

## Conclusions

The high prevalence of hypertension in Lusaka calls for an integrated and comprehensive public health approach to ameliorate the inevitable consequences. Taking into account the observed associations of the risk factors, targeted prevention and control measures such as health education and structural interventions guided by a strategic plan on non-communicable diseases ought to be instituted. These must include increasing opportunities for exercise by environmental engineering, food labelling for lipids and salt content, anti-smoking legislation and alcohol abuse reduction strategies.

## Competing interests

The authors declare that they have no competing interests.

## Authors' contributions

FMG participated in the design of the study, supervised data collection and helped to draft the manuscript. SHN, OB, PS and CZ participated in the interpretation of the results and helped to critically revise the manuscript. ER participated in the interpretation of the results and participated in drafting the manuscript. SS participated in the design of the study, supervised data collection, performed the statistical analysis, participated in the interpretation of the results, and participated in drafting the manuscript. ASM participated in the interpretation of the results and led the drafting of the manuscript. All authors read and approved the final manuscript.
